# The Effect of the Surface Area of Carbon Black Grades on HNBR in Harsh Environments

**DOI:** 10.3390/polym11010061

**Published:** 2019-01-04

**Authors:** Winoj Balasooriya, Bernd Schrittesser, Gerald Pinter, Thomas Schwarz, Lucia Conzatti

**Affiliations:** 1 Polymer Competence Center Leoben GmbH, Roseggerstrasse 12, 8700 Leoben, Austria; Bernd.Schrittesser@pccl.at; 2 Department of Polymer Engineering and Science, Montanuniversitaet Leoben, Otto Glöckeltrasse 2, 8700 Leoben, Austria; Gerald.Pinter@unileoben.ac.at; 3 SKF Sealing Solutions Austria GmbH, Gabelhoferstrasse 25, 8750 Judenburg, Austria; Thomas.Schwarz@skf.com; 4 National Research Council, ISMAC Genova, Via De Marini 6, 16149 Genova, Italy; Lucia.Conzatti@ge.ismac.cnr.it

**Keywords:** oil and gas, elastomers, HNBR, carbon black, rapid gas decompression

## Abstract

Concerning the still rising demand for oil and gas products, the development of new reliable materials to guarantee the facility safety at extreme operating conditions is an utmost necessity. The present study mainly deals with the influence of different carbon black (CB) filled hydrogenated nitrile butadiene rubber (HNBR), which is a material usually used in sealing applications, on the rapid gas decompression (RGD) resistance in harsh environments. Therefore, RGD component level tests were conducted in an autoclave. The supporting mechanical and dynamic mechanical property analysis, the microscopic level investigations on the material and failure analysis were conducted and are discussed in this work. Under the tested conditions, the samples filled with smaller CB primary particles showed a slightly lower volume increase during the compression and decompression phases; however, they steered to a significantly lower resistance to RGD. Transmission electron micrographs revealed that the samples filled with smaller CB particles formed larger structures as well as densified filler networks including larger agglomerates and as a consequence a decrease effective matrix component around the CB particles. Apparently, at higher loading conditions, which already deliver a certain level of mechanical stresses and strains, the densified filler network, and especially a lower amount of effective matrix material composition, adversely affect the RGD resistance. SEM-based fracture analysis did not identify any influence of the CB grades tested on the crack initiation site; however, it revealed that the cracks initiated from existing voids, hard particles, or low strength matrix sites and propagated to the outer surface.

## 1. Introduction

The durability and the performance of elastomers are greatly affected by the working environment. Especially in harsh conditions, for example, elastomeric materials are exposed to high pressure, high temperature, and different media, and as a result, their properties deteriorate significantly and even component failure occurs [1]. Therefore improving the material properties to face the challenges in use are of utmost interest among materials scientists. The specific catastrophic failure phenomenon, the rapid gas decompression (RGD), which occurs in almost every fluid-handling elastomeric component as a result of sudden pressure release, is well-known, but is still under discussion even after decades [2,3,4,5,6,7,8,9]. This failure leads to high volume increase, crack initiation, crack growth, and the complete destruction of the component. The whole process can be divided into the pressurization and the depressurization phases. The first occurs due to the penetration of different media (mainly in gas conditions) at high-pressure and high-temperature atmospheres; it leads to a volume increase, strongly depending on the temperature, the pressure, and the media used [6,9,10]. The latter is a highly complex phase, where a high volume change of the component due to the pressure reduction occurs at ambient conditions. A stress-state change is expected in nominal stresses from compression to tension during the decompression cycle. The high volume change of gas leads to a three-dimensional pneumatic tension state in the material and it creates an additional and complementary assumed uniform body stress in the sample [2,6,11]. The compressed gas nucleates at voids or loosely bonded filler/rigid inclusion surfaces during rapid gas decompression; the voids inflate resulting in tensile stresses or strains in the void walls. This can lead to surface blisters or cleavage cracks, according to the permeability of gases into the material and induced stress levels [2,12].

Furthermore, during the compression phase, with high-pressure gas exposure, mainly two effects can be expected: (i) the plasticization of the polymer matrix leading to increased free volume and subsequent backbone movement and (ii) the compression of the polymer matrix. The first occurs mainly with highly soluble gases with a high boiling point (e.g., CO_2_) and the second mainly with gases with lower boiling points (e.g., N_2_). The nature of the gas and the applied pressure define the dominance of these opposite effects in the material and lead to a change of the mechanical and thermal properties [4]. Therefore, the permeation and solubility of gas strongly influences the material behavior. Additionally, the interacting media has a significant effect as well. Many previous research efforts could be found in the literature dealing with the media and permeation processes [8,13,14]. Especially, carbon dioxide (CO_2_) can become a supercritical fluid and therefore a good solvent at relatively moderate pressure and temperature [15]. This concerns the elastomeric components in use when CO_2_ is present. Especially in the oil and gas fields, the existence of a moderate amount of CO_2_ in the reservoir can induce a failure in elastomeric seals that otherwise perform well in high-pressure gases [15]. This is because carbon dioxide has a small, permanent dipole with uneven charge distributions and it is capable of multiple types of associations, especially with polar elastomers. For example, in CO_2_, the swelling is higher for fluoroelastomer (FKM) and hydrogenated nitrile butadiene rubber (HNBR) compared with ethylene propylene diene monomer (EPDM) [4,10,16].

The RGD phenomenon of elastomers is common in harsh environments and this rather complex process is seemingly a combination of many factors. Several authors have made efforts to identify the RGD behavior in different aspects dealing with the influence of testing parameters, fracture mechanical approaches and simulation models [2,3,4,5,6,7,8,9,10,11]. However, it is still of prime interest to identify the influence of the material composition, especially of the reinforcing fillers. Carbon black (CB) is an essential ingredient of most rubber formulations and has a considerable influence on the performance of the final product. This is especially true for RGD behavior, including the high probability of crack initiation in rubber matrix–filler interface, and considering the space between particles where the matrix is highly constrained [3]. Hence, it is vital to understand how the RGD and the related performance of the materials are affected by the nature of the CB grades used. Therefore, the aim of this research work is to consider mainly the RGD performance of HNBR filled with different CB grades in contact with CO_2_ in conditions near the service level and to delve more deeply into the reinforcement mechanism of the CB filler system.

The use of CB in elastomers is common to achieve a reasonable range of mechanical properties required for a great variety of modern applications. It increases stiffness, modulus, rupture energy, tear strength, tensile strength, cracking resistance, fatigue resistance and abrasion resistance [17,18,19]. At the beginning of the 19th century, CB was accidentally identified as a substance to eliminate the inherent stickiness of rubber after zinc oxide had long been used [20]. CB as a reinforcing filler also reduces the cost of the end product and modifies the electrical and optical properties of the polymer matrix and is one of the most stable chemicals [17,19,20].

Considering CB as a reinforcing media, it relies on several factors, i.e., the primary particle size (specific surface area), the structure (the degree of irregularity of the filler unit) and the surface activity [18,19,20]. These factors evolve from the source or processing characteristics, and they are named accordingly; for example, furnace black, lampblack, thermal black, acetylene black, and channel black [20,21]. ASTM nomenclature uses the letter “N” to indicate a normal curing rate of typical furnace black which has had no special modification to alter the influence of the curing rate of rubber, followed by three numbers indicating the reinforcing character [20,22]. The first number refers to the average typical size of the elementary particle size. Therefore, a lower number refers to a lower particle diameter and higher surface area, for example N550 has a higher primary particle size and a lower surface area compared with N330 or N110 [21]. The last two digits of the number refer the structure of the aggregate and its reinforcing character, for example, N340 is a better reinforcing grade compared with N327 while in the same primary particle size diameter range [21,22]. The primary particles do not exist as separate particles, but they are fused together to form aggregates and this three-dimensional arrangement designates the “structure” of the CB grade [20,21,22]. A high degree of branching or clustering of the aggregates characterizes a “high structure”, and vice versa [20,23]. Additionally, the branched aggregates create porous structures and a higher surface area compared with the compact dense spherical particle structures. This creates additional filler surfaces available to interact with rubber and results in better reinforcing capabilities [21,23,24].

With regards to the particle size, particles up to 100 nm diameter are responsible for the reinforcement, irrespective of the particle structure [22]. However, particles larger than 10^3^ nm are hardly responsible for reinforcing effects but used to increase viscosity by a hydrodynamic effect [23]. A smaller particle size, which gives higher surface area, manages to create a higher degree of cross-links with the elastomer and has a positive effect on the tensile strength, elastic modulus, the hardness of the elastomers as well as the compression set [19,23,24]. Mainly the filler–rubber cross-links are of a physical nature (physisorption), but the surface chemistry of CB has an effect on the vulcanization behavior of filled compounds [18]. Therefore, it is generally accepted that the surface area has a significant effect on increasing vulcanization reactions and mechanical properties [17,19].

The anisometric nature of the aggregate has a progressive influence on creating the entanglements of rubber molecules with the CB and their attachment (mechanical interlocking) [19]. Furthermore, the primary particle size correlates to the structure, for example, smaller primary particle size CB grades tend to create higher structural CB aggregates and vice versa [23]. A low structure may contain less than 20 primary particles per aggregate; a high structure may consist of up to 200 particles [21]. The aggregate is the smallest form of a given CB grade because they flocculate together to form weak, giant secondary aggregates called agglomerates [22,23]. There are two models to explain these loosely bound filler networks: (i) the filler–filler physical van der Waals interactions [18] and (ii) the attachment through a polymer layer, which is in nanometer-scale, in between two attractive filler surfaces [25]. In the latter, the polymer chains, which are in confinement, are strongly immobilized and these bonds create glassy-like bridges between the filler aggregates and transmit the stresses along the filler network [26].

When an uncured elastomer is blended well with a CB grade for a period, the elastomer chains are bound to the carbon black aggregates through different mechanisms, i.e., chemical/physical bonds, immobilized chains creating glassy-like bridges in-between fillers or the mechanical inter-locking of elastomer chains around/through the filler surfaces. This creates highly immobilized and localized rubber chains forming a rubber shell surrounding the CB aggregates [19]. This highly restricted rubber layer is no longer a part of the elastically active rubber matrix, but a part of the filler network and it increases the effective filler volume [19,24].

**Figure 1:**
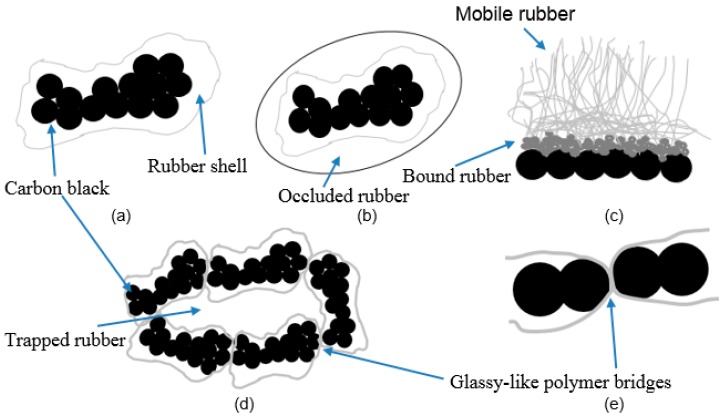
Models of filler–rubber interaction mechanisms and behavior of rubber: (**a**) strongly bound rubber around the carbon black; (**b**) occluded rubber around the rubber shell; (**c**) bound rubber near the carbon black (CB), transition layer, and then the mobile rubber far from the CB surface; (**d**) CB agglomerate and trapped rubber in between the aggregates; (**e**) filler aggregates are bound through glassy-like polymer bridges [18,19,24].

In Figure 1, certain models explaining the filler–rubber interaction are summarized. Figure 1a shows the stronger bonding of rubber around the filler aggregates to form a rubber shell on the surface, the so-called “bound rubber”, with increasing stiffness when it is closer to the CB and vice versa. When irregular filler structures exist, the rubber occludes between the inner voids of irregular contours and forms an outer layer called “occluded rubber” as shown in Figure 1b. It is not strongly bound like the rubber shell, but experiences reduced mobility. As shown in Figure 1c, this bound rubber is strongly attached to the CB closer to the surface; away from the surface, the mobility of rubber is increased. Figure 1d shows a possible cluster mode of fillers, creating filler agglomerates, which trap some rubber excluding from the rest of the matrix. This is called “trapped rubber” and it becomes ‘free’ once the aggregate structure breaks apart during the deformation [17,24]. The bound rubber and the occluded rubber content depend on several factors, i.e., CB loading, CB surface area, structure, temperature, and the dwell time of CB in the rubber before curing [24]. A model to explain the filler–filler bonds to create filler agglomerates is illustrated in Figure 1e, where some rubber molecules remained in-between filler particles and these behave like glassy-like polymer bridges.

The filler aggregates and agglomerates in CB grades are responsible for the unique characteristics of filled elastomers. However, when CB is mixed with a rubber in a mill, the encountered shear forces could damage the aggregates and agglomerates reducing their sizes and structures [26]. In addition, many researchers assumed the well-known elastomeric phenomenon, Mullin’s effect and Payne’s effect, a result of the breakdown and re-aggregation of softer filler agglomerations [17,27,28]. According to these models, the tension and relaxation in a cyclic loading condition lead the dynamic breakdown and the re-aggregation of filler–filler bonds, especially the glassy-like polymer bridges between filler aggregates. The damage to the bonds is structurally reversible, but the re-aggregation of filler particles takes place resulting in hysteretic effects because the re-aggregated polymer bridges do not recover to the same strength as unbroken bridges, leading to a softening of the filler–filler bonds [25]. Furthermore, some researchers discussed the effects of CB in rubber materials; for example, Zhang et al. [29] investigated the effect of the particle size distribution of CB on the mechanical properties, and did microanalysis of the fractured sample of vulcanized NR with high abrasion furnace (HAF). They observed a better particle distribution with increasing emulsifiers in the material and enhanced mechanical properties with higher filler dispersion in the rubber matrix due to the better interaction of carbon black and rubber matrix [29].

## 2. Materials and Methods

The influence of CB grades in HNBR on the RGD resistance was tested at component level. The macroscopic and microscopic aspects of the material behavior were observed. The material compositions, the testing methods, and the specific settings are described below in the following section.

### 2.1. Materials

An HNBR (ACN 36%) grade, peroxide cross-linked, stabilized with anti-oxidants, was selected for the investigations. The manufactured compound was provided as disc plates and cylinders for investigations. In this study, different CB grades, which contained large, middle and small primary particle size CB in different compositions to form 85 phr filled HNBR, as shown in Table 1. The large, middle and small size of CB represent the CB with roughly 50, 100 and 150 mg/g of iodine adsorption number, respectively. The names of exact CB grades are not mentioned here due to confidentiality constraints. The different grades have the similar recipe except the mentioned CB grade difference and they were added to the matrix in an internal mixer. The similar amount of loading of different CBs was selected to ensure a range of particle sizes, surface areas, and structure.

The specimen dimensions and test parameters are summarized below.

### 2.2. Rapid Gas Decompression Test

The component level-RGD tests, which indicate the bulk deformation behavior of HNBR under highly confined conditions, and study crack initiation and crack growth under high deformation rates, were carried out with a high-pressure autoclave test system (SITEC, Sieber Engineering AG, Zürich, Switzerland). The cylindrical specimens were prepared by machining to obtain the shape of the cross-section of an O-ring, maintaining 8 mm in both diameter and height. The specimens were placed inside the autoclave in a freestanding position (unconstrained). The system was pressurized up to 150 bar with pure CO_2_ at 90 °C. After ~22 h, it was depressurized to study the decompression failure with a rate of 100 bar/min. This high decompression rate, compared with the proposed rate in the standard [30], achieves a condition near service level for the elastomeric materials. The sensors continuously measured the pressure and the temperature inside the autoclave. A camera, which was situated outside the autoclave, monitored the in-situ volume expansion of the test specimens through the glass window, and later the volume change was calculated with the assistance of Tracker software (Tracker 4.95, Douglas Brown physlets.org/tracker). This experimental set-up is further explained in the previous research work [6,9,31]. The failure analysis of the tested samples was conducted utilizing a light microscope and scanning electron microscope. Every tested cylindrical specimen was examined at three radial cut sections by light microscope for internal cracks and every material grade test was repeated with three specimens; the crack size and the number of cracks rated the material according to the NORSOK ranking (Table 2).

### 2.3. Tensile Test

The tension tests on dumbbell-shape specimens (S2) were carried out according to the DIN 53504 standard. The specimens were punched out from the ~2 mm thick plates maintaining a width of 4 mm between the shoulders. All tests were performed with a Zwick universal testing machine (Zwick Roell, Test expert, Ulm, Germany) using a 1 kN load cell, at a constant crosshead speed of 200 mm/min with an initial gauge length of 43 mm. Five specimens of each grade were investigated to ensure the reproducibility.

### 2.4. Dynamic Mechanical Analysis

The microstructures of differently filled HNBR were investigated using DMA measurements and the experiments were conducted with a DMA 861/40N testing device (Mettler Toledo GmbH, Schwerzenbach, Switzerland). All tests were carried out within the temperature range of −50 °C to 100 °C using liquid nitrogen as a cooling agent with a frequency of 2 Hz and a heating rate of 2 K/min. The parallel parts between the shoulders of the dumbbell specimens (thickness of 2 mm and width of 4 mm) were used as the specimens in tension mode with a clamping distance of 19.5 mm.

### 2.5. Scanning Electron Microscope (SEM) Analysis

The fracture surfaces of RGD tested samples were observed utilizing an SEM (Tescan VEGA-II, Brno, Czech Republic). The broad and magnified views of surfaces helped to identify the crack initiation and propagation in different material grades and different fracture behaviors based on different CB compositions.

### 2.6. Transmission Electron Microscope (TEM) Analysis

The TEM micrographs analysis was performed for all four material grades utilizing a Zeiss EM 900 microscope (Carl Zeiss AG, Oberkochen, Germany) with an accelerating voltage of 80 kV. Ultrathin sections (~50 nm thick) were prepared by using a Leica EM FCS cryo-ultramicrotome at −100 °C.

## 3. Results and Discussion

In the following section, the influence on the RGD behavior of HNBR samples filled with different CB grade compositions is compared. It is vital to understand this complex phenomenon by the influence on macroscopic properties and the micro/nano level observations from different CB grades. Therefore, the CB primary particle/aggregate sizes and their distribution are investigated with TEM micrographs. The monotonic loading tensile properties and dynamic mechanical properties are characterized identifying the macroscopic behavior. The possible microstructural morphologies involved with the RGD damage mechanisms based on the SEM images are discussed as well.

### 3.1. Morphological Investigations of CB Filled Samples by TEM

**Figure 2:**
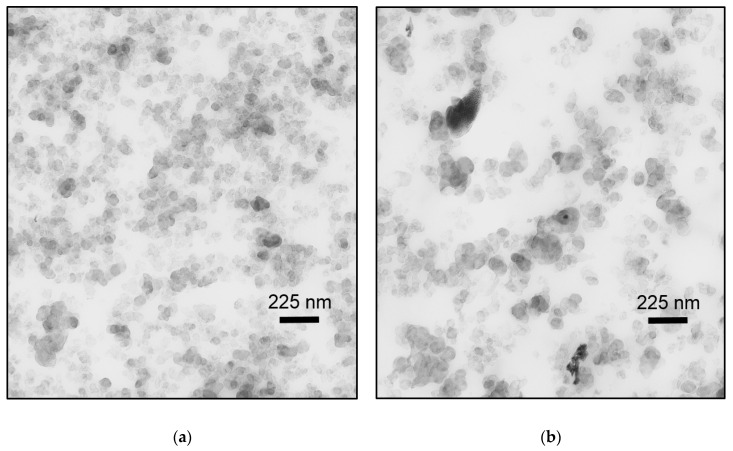

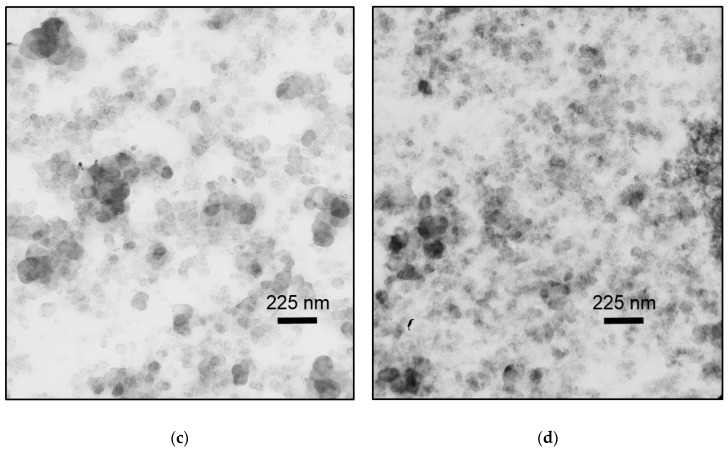
TEM micrographs of HNBR filled with different CB grades: (**a**) HNBR1; (**b**) HNBR2; (**c**) HNBR3; (**d**) HNBR4.

Composite structures were observed by TEM micrographs at different magnifications analyzing the CB filler distribution, structure, and network behavior. The representative micrographs for the different material grades are shown in Figure 2, where, a, b, c, and d represent HNBR1, HNBR2, HNBR3, and HNBR4, respectively, with the same magnification. It should be mentioned that at low magnifications, all grades showed relatively homogenous distribution within the elastomer matrix and seemingly, the processing method did not influence the distribution of different CB grades in the conditions tested. Therefore, only the higher magnification micrographs are shown here for comparison purposes. The dark objects represent the CB and the light color areas represent the HNBR matrix material. Pseudo-spherical shaped primary particles could be identified in every sample, while several aggregations of filler particles are formed in the rubber structure, as those sizes are somewhat larger than the primary particle sizes.

As shown in Figure 2a, HNBR1 shows an even distribution of CB aggregates and a well-connected network throughout the matrix. The diameter of the majority of primary particles is in the range of ~30 nm and they are clearly packed creating irregular-shape structures which are connected each other. In Figure 2b, HNBR2 shows a rather disconnected network and more matrix material around and between CB aggregates. In fact, most primary particles in HNBR2 are bigger (~50 nm) than in HNBR1. Therefore, HNBR2 contains rather isometric CB structures, which are a few bigger particles formed together with less random branching, compared with HNBR1. In Figure 2c, small and big primary particles are clearly visible roughly in similar amounts. Further, the CB particle network density is higher compared with the HNBR2, however lower compared with the HNBR1. The number of particles in the structure and the branched nature as well as the matrix material around the aggregates is in moderate range compared with HNBR1 and HNBR2. With reference to the HNBR4 as shown in Figure 2d, the CB primary particles are relatively smaller (~15 nm), the aggregates are bigger and the network is much denser compared with the other tested grades. The aggregates have apparently formed larger agglomerates, which are indicated as dark clouds in three-dimensional form. Therefore, higher filler–filler interactions and relatively less matrix material around the filler particles are observed.

### 3.2. CB Grade Influence on Dynamic Mechanical Properties

**Figure 3:**
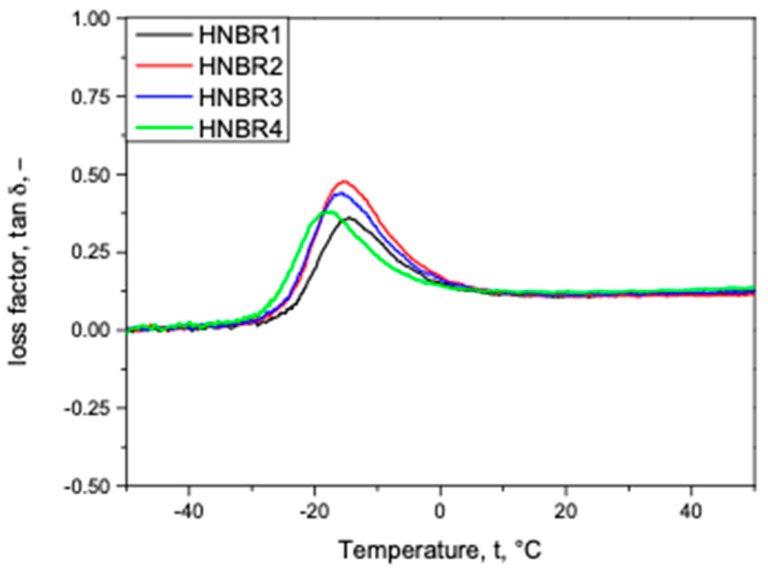
The temperature dependence of different CB grade compositions in HNBR on DMA analysis: the loss factor tan δ.

The influence of the different CB grades on the mechanical properties of rapidly varying stress conditions over a range of temperature (−50 °C to 50 °C) was studied. The CB grade dependence on the loss factor (tan δ) deviation along the temperature is shown in Figure 3.

As depicted in Figure 3, the peak of the loss factor, which correlates to the glass transition temperature (T_g_), and the intensity and width of the loss factor peak, which imply the damping properties [32], are slightly different in every tested grade. The lowest T_g_ was recorded with HNBR4, while HNBR2, HNBR3, and HNBR1 displayed slightly higher T_g_ values, respectively. However, a different trend in damping properties was witnessed, for example, the highest damping in HNBR2, and lower damping properties in HNBR3, HNBR4, and HNBR1, respectively. The storage modulus deviation, the glass relaxation onset and the reduced magnitude followed similarly to the loss factor deviation trend. Therefore, it is not shown here.

According to the literature, the higher filler surface and the filler structure offer better filler–rubber interactions as a result of strong bound and occluded rubber layers, where the chain motion is more restricted compared with the rest of the matrix [24]. Consequently, the loss of segmental mobility increases the T_g_ and damping properties [24,33]. Therefore, the ranking of HNBR2, HNBR3, and HNBR1 for having lower damping properties and higher T_g_, respectively, correlate with the relatively higher filler surface area of CB in the composition and their better filler structures. Similarly, the TEM micrographs showed that HNBR1 contained the best filler network, while HNBR3 and HNBR2 contained comparatively less densified filler networks, respectively. However, HNBR4, which accommodates the highest primary particle surface area among the CB grades, does not correlate the trend with other samples. As shown in Figure 2d, HNBR4 contains the highest filler network and larger agglomerates in the material. Therefore, the filler agglomerates in the material reduced the possible filler–rubber interactions and decreased the possible reinforcement and this could be associated with the lowest T_g_ and moderate damping properties of HNBR4. However, the slightly enhanced damping properties could be associated with the filler–filler interactions. The nominal hardness values for the tested grades showed as 87.3, 85.3, 86 and 86.3 SHA, for HNBR1, HNBR2, HNBR3, and HNBR4, respectively, and it is also in good agreement with the damping properties revealed in DMA. Theoretically, the rigid rubber shell due to the higher surface and better filler–rubber interactions enhances the hardness of the total composite [19].

### 3.3. CB Grade Influence on Tensile Properties

**Figure 4:**
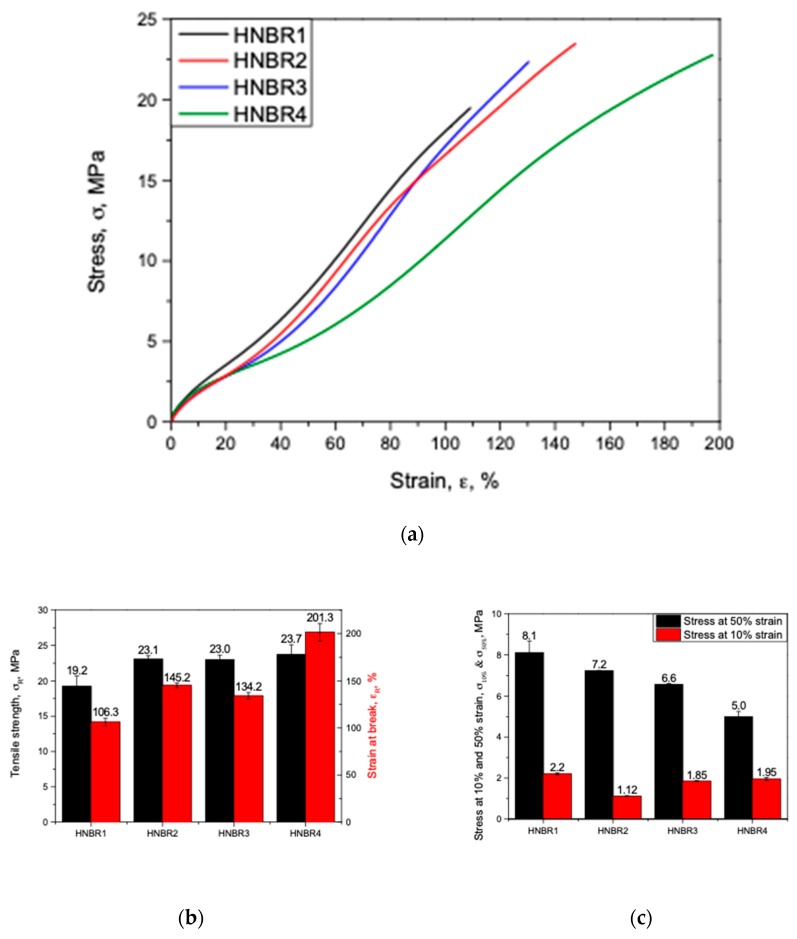
Tensile properties of CB-filled HNBR: (**a**) typical stress–strain curves; (**b**) tensile strength; (**c**) stress at 10% and 50% strain.

The influence of the filled CB grades in HNBR in monotonic loading conditions is compared in Figure 4, where the stress–strain deviation, the tensile strength (σ_R_) and the strain at break (ε_R_), the stress at 10% (σ_10%_) and 50% strain (σ_50%_) deviation in different samples are shown in Figure 4a–c, respectively.

As shown in Figure 4a, a unique stress–strain behavior for the differently filled HNBR was apparent. Especially, HNBR1 shows higher stresses from the beginning of the tests; however, the HNBR2 and HNBR3 deviate with slightly lower values, but in a similar range of properties. HNBR4 behaves differently from the rest, with rather high stresses at the beginning of the test, but showing rather softer material properties thereafter ~10–15% of strain. As depicted in Figure 4b, HNBR1 shows the lowest σ_R_, and HNBR2 reveals relatively higher σ_R_, while HNBR3 lies in between them. Additionally, the HNBR4 samples demonstrate the highest σ_R_ values among the tested samples. The trend was similar for the strain at break, ε_R_. A relatively different behavior for the σ_10%_ and σ_50%_ was observed, as shown in Figure 4c, where, HNBR1 shows the highest stress levels in 10% strain, HNBR2 has the lowest σ_10%_ and HNBR3 remains in-between them, while HNBR4 owns the second best σ_10%_ after HNBR1. However, σ_50%_ reveals a different trend, where HNBR1 has the highest values and HNBR2, HNBR3 and HNBR4 have lower values, respectively.

Theoretically, the higher surface primary particles tend to form higher structures and subsequently to deliver higher tensile properties [19,23]. However, some researchers experienced different tendencies based on achieving the balance between the CB loading and agglomerates [23]. HNBR1, which has a higher degree of CB with higher surface in the composition, shows a rather brittle nature, with the highest stiffness and lowest strain at break behaviors, compared with HNBR2 and HNBR3. Similarly, the moderate tensile properties of HNBR3, in-between HNBR1 and HNBR2 could be considered as the effect of the amount of CB with higher filler surface in the composition. Additionally, HNBR4, which contains the CB with highest surface area in a higher degree in the composition, shows the highest strength at break, but a softer material behavior compared to other material grades. The TEM micrographs, as shown in Figure 2, perhaps confirm this behavior, as HNBR1 has better-networked, anisometric shaped filler aggregates, while HNBR2 and HNBR3 possess these to the least and moderate degrees. Therefore, the higher primary particle surface area, or structure, has shown an influence to a certain level, even in these highly filled conditions. The irregular shaped structures are expected to increase the possible elastomer–rubber interaction and as a result larger bound and occluded rubber layers around filler. These layers are assumed to be partially restrained from deformation, and this consequently increases the effective filler concentration [18,19,24,33]. Therefore, the whole composite has increased stiffness, owing to the decreased segmental mobility of polymer chains and consequently a reduction of the flexibility of the rubber matrix [18,33]. However, in HNBR4, which contains a higher amount of highest surface area CB, apparently form aggregates and larger agglomerates (as Figure 2d revealed). As shown in Figure 1, these can probably create more filler–filler interactions and decrease the matrix material around the particles. By applied external loading, the weakly bonded filler–filler agglomerates tend to collapse and expose the trapped rubber it may contain. Additionally, at higher stresses, the broken filler agglomerates slightly restrict the free movement of elastomeric chains [18,33]. Therefore, at lower strain levels, relatively higher stress values but at higher strain levels, a softer material behavior and relatively higher strain at break values can be expected in HNBR4.

### 3.4. CB Effect on Rapid Gas Decompression

**Figure 5:**
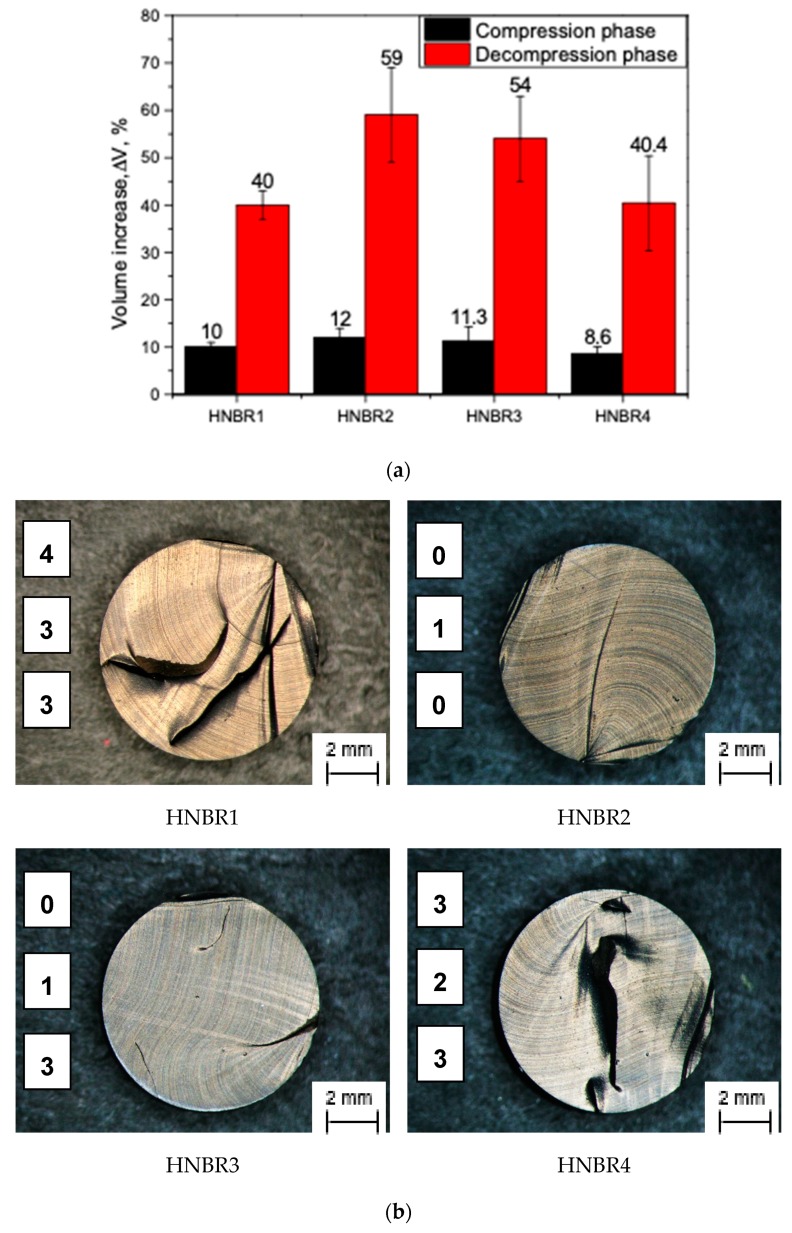
Influence of CB grades composition on rapid gas decompression (RGD) performance of HNBR: (**a**) volume increase during compression (ΔVC) and decompression (ΔVD); (**b**) visual observation of cylindrical specimens and the NORSOK material ranking (every number on the right represents a different measurement).

The RGD test data from the autoclave tests were reduced to two specific values; the maximum observed volume change during the compression phase (the difference of volume at saturation phase to initial volume) (ΔVC), and the maximum observed volume change during the decompression phase (volume expansion compared with initial volume) (ΔVD). The behavior of ΔVC and ΔVD for different material grades is as summarized in Figure 5a. The optical microscope pictures for the observed cracks in the cylindrical specimens of the different material grades are shown in Figure 5b. The numbers in the microscope picture indicate the NORSOK rating, measuring the number of cracks and intensity of cracks, of the implemented measurements for every material grade.

As depicted in Figure 5a, tested samples reveal slightly different volume increases to each other during the compression phase; HNBR2 and HNBR4 possess the comparatively highest and lowest volume increase in this small range, respectively. Considering the decompression phase, HNBR1 and HNBR4 demonstrate a lower volume change (~40%), while HNBR2 reveals the highest volume change (~60%), and HNBR3 (~54%) shows a moderate increase. However, it should be mentioned that all tested specimens for HNBR4 fell from view from the observation window of the autoclave after a certain time during the decompression phase with a fast crack growth from the inside to the outside of the specimen. All other specimens remained in their freestanding position with only slight movements. Therefore, ΔVD of HNBR4 samples may not indicate the precise values or hold high scattering. Despite a certain tendency in the ΔVC and ΔVD, the microscope observation and the NORSOK ranking indicate a different behavior. A higher number of cracks were apparent on the cross-sections of HNBR1 and HNBR4; they were considerably more than 80% of the cross-section. Therefore, the lower resistance to RGD was observed with HNBR1 (4, 3, 3) and HNBR4 (3, 2, 3). While HNBR3 (0, 1, 3) and HNBR2 (0, 1, 0) revealed only a few and minor cracks in the cylindrical specimens, respectively, and consequently a better resistance to RGD failure.

As depicted in Figure 5a, the ΔVD follows a trend similar to ΔVC, but in a greater magnitude. The volume increase during the high-pressure CO_2_ exposure could be explained as a parameter of solubility of the gas into the material and the dissolved gas in the material as responsible for volume expansion during the decompression phase. In the tested conditions, the CB grades with the different surface area have seemingly influenced the solubility of the gas. For example, in HNBR1, HNBR2, and HNBR3, the ΔVC and ΔVD show a correlation with the CB grade, where a higher degree of middle range primary particles in the composition has caused a comparatively lower volume increase. Similarly, HNBR4 has the lowest volume change during compression among the tested samples, where higher degree of small particles are contained. In general, higher gas solubility and a lower rate of diffusion in elastomers tend to deliver a lower RGD resistance in the material [9,34], however, a clearly improved RGD resistance in HNBR2 and HNBR3 was observed compared with the other two materials, irrespective the higher compression and decompression volume increases.

At high-pressure exposure, gas permeates into the elastomers and dissolves in the matrix material at small imperfections or filler–matrix interfaces, if the filler–matrix interaction is poor, until saturation [4,6,9,31]. In the tested conditions, where the samples contained the same filler loading, the HNBR with lower surface CB showed a slightly higher volume increase in the compression phase, ΔVC. Briscoe et al. [4], explained the addition of filler into matrix alter the sorption properties based on the quality of the filler–rubber interaction. If the interface is strong, it significantly reduces the gas sorption compared with unfilled and vice versa. This is attributed as the filler–rubber interaction creates strong rubber layers around the filler and it decreases the effective matrix amount, which can possibly dissolve the gas. On the other hand, filler inclusion improves the mechanical properties of the matrix and it reduces the entering gas into the material [4]. Similarly, Jamabe et al. [7] explained, that gas solubility of filled rubber is influenced by surface area of the filler as well as the interface structure of filler–rubber interaction. However, in their experiments with CB filled EPDM in high-pressure (10 MPa) hydrogen atmosphere, they observed that, at lower filler loading levels (25 phr), CB grades with smaller primary particles had a higher solubility, but at higher loading levels (50 phr), they did not find a clear trend [7]. Under the tested conditions, higher surface CB tends to create complex structures (also visible in TEM micrographs in Figure 2); it could deliver better filler–rubber interactions, enhance the effective filler concentration through possible restricted matrix movements (Occluded and bound rubber layers), and consequently, reduce the elastically effective matrix material component. This can be a plausible interpretation for the ΔVC trend. Further, the mechanical properties, especially the trend of σ_10%_ of the quasi-static tensile test results (Figure 4c), have a possible influence on the same trend of ΔVC. The volume increase in compression and decompression phases (ΔVC and ΔVD) deviate in less than 10 percent of strain levels (<10%, ε), therefore σ_10%_ is in good agreement with ΔVC and ΔVD. Similarly, the DMA damping properties deviated in a similar order. Therefore, apparently the solubility of gas is slightly decreased due to decreased effective matrix content and increased mechanical properties in higher filler surface conditions.

During the decompression phase, the accumulated gas in the material is subjected to nucleate at expansion-forming micrometer-sized bubbles. Initially, they do not form cracks so long as the material deforms elastically without inhibition [2,4,12]. If no crack was initiated, the dissolved gas becomes extinct desorbing them without any visible traces [8]. However, if the high rate of elastic expansion meets a low strength matrix or hard body inclusions where the stress concentrates, crack initiation and propagation take place in the elastomer bulk as well as in the hard body-matrix interfaces [6,35]. In the tested conditions, the volume increase during the decompression, ΔVD, (Figure 5a) seemed to follow the same trend of ΔVC and it is probably correlated to the stiffness of the different grades and the degree of dissolved gas in the saturated state. Additionally, ΔVD could be reduced by possible cracks propagated during the decompression phase. It is generally accepted that the resistance to RGD of elastomers is increased if the gas sorption into the material is lower and the physical properties (stiffness, tensile strength, tear resistance, etc.) of the material are higher [6,7,8,9,31,35]. However, NORSOK ranking of tested samples revealed otherwise: where HNBR1 and HNBR4 have the lowest RGD resistance, while HNBR3 and HNBR2 reveal a significantly better resistance to RGD. The RGD phenomenon of elastomers is a rather complex process with a combination of many factors. However, in this test series, the samples are highly filled, consequently an enhanced filler network and agglomerates as well as reduced matrix component are expected. As revealed in TEM micrographs (Figure 2), the networks of CB in all four material grades are clearly influenced by the higher particle surface. The higher CB structures, which were formed due to higher surface area, tend to form thick rubber layers around them, which enhance the effective filler amount and decrease the active matrix component. However, HNBR4, which contains CB with the highest surface area, apparently creates larger filler agglomerates and less matrix material around fillers. In these highly filled conditions, the higher surface area CB is adversely effective in RGD resistance; HNBR2, which has the lowest structure and CB network density, reveals the best RGD resistance, while HNBR3 and HNBR1 disclose relatively lower RGD resistance. HNBR3 and HNBR1 contain comparatively higher CB structures and networks in the materials, respectively. Therefore, it could be assumed that if there were a sufficient amount of elastically active matrix material in the material, the bubbles nucleation and growth during the decompression phase would be assisted. However, when the filler network is densified or effective CB volume increased due to the rigid matrix around the CB, subsequently the active matrix composition is decreased and the possible bubble growth in matrix hinders during decompression phase. This concentrates stresses around the rigid inclusions and tends to initiate cracks.

HNBR4 at lower strain levels maintains relatively higher stress values due to filler–filler interactions. However, above a certain strain level, the agglomerates tend to break and steer to a softer material behavior. Therefore, the lower ΔVC values were observed and at the same time, a lower RGD resistance was experienced. This material grade even fell from sight from the observation window during the autoclave test: the sample, which was in a freestanding position on the ground displaced, as a result of faster crack growths in the material. The stress concentrations during the decompression volume expansion probably tend to break the CB agglomerates and once the crack initiates, it grows faster through the rather soft material.

CB loading and CB surface area improve the mechanical properties in elastomeric composite, which are essential for RGD resistance. However, apparently, after a certain level of CB loading, it is better to have lower CB surface/bigger primary particles in the material in terms of RGD resistance.

### 3.5. SEM Based Fractography

**Figure 6:**
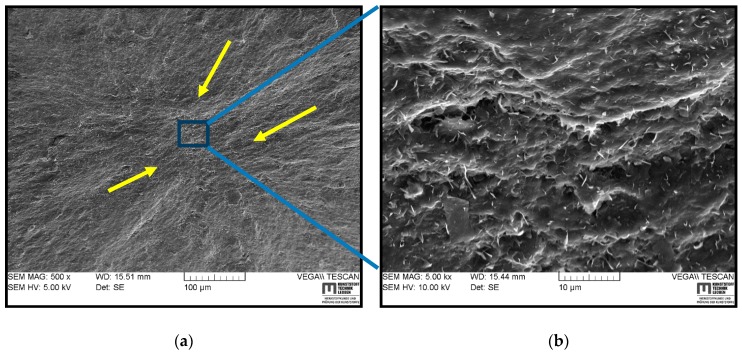
SEM images of a typical fracture surface of HNBR1 in different magnifications: (**a**) fracture initiation and propagation; (**b**) magnified view of fracture initiation.

In the following section, the fracture surfaces of RGD tested cylindrical specimens, which were examined using SEM, are displayed for understanding the crack initiation, the modes of crack propagations and the morphology of rubber/CB of differently filled samples. Figure 6 shows the SEM images of a fracture surface of HNBR1 on a more global scale (a) and the magnified view of the crack initiation (b). The point where many small tearing lines converge, as highlighted in Figure 6a by arrows, can identify the crack origin. The crack growth lines seem to create a microscopically rough surface. Therefore, the fracture surface texture of the HNBR1 suggests that it contains pits/cavities as a result of reinforcing CB agglomerates coming out of the matrix surface. As shown in Figure 6b, no real pre-existing cracks or defects were apparent in the vicinity of the initiation point, but only a rough surface as a result of loosened agglomerate detachment. The literature suggests that the loosened agglomerates, due to the inhomogeneous cross-link density, can act as stress raisers and offer a path for the tear to follow [7,12]. However, this seems like a stable crack growth as the fracture surface has a similarly rough texture along the crack growth.

**Figure 7:**
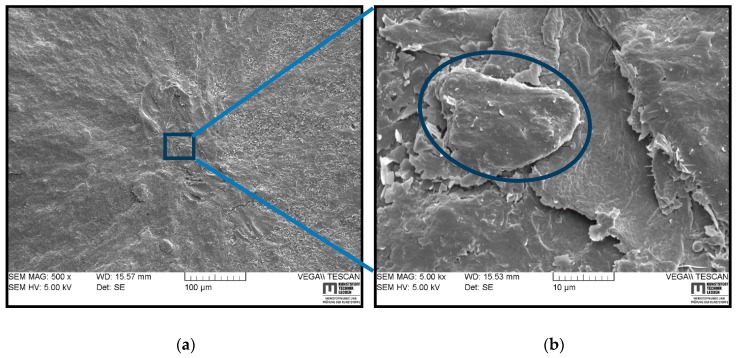
SEM images of a typical fracture surface of HNBR2 in different magnifications: (**a**) fracture initiation and propagation; (**b**) magnified view of fracture initiation from a low strength site.

Figure 7 shows a fracture surface of HNBR2 in different magnifications. A crack initiation and propagation sight are indicated in Figure 7a, while Figure 7b focuses on the initiation point. Similar to Figure 6, the crack initiation is clearly visible in Figure 7a, where many small tear lines spread out from one place. As visible in the magnified view in Figure 7b, the crack initiated from a rather flat surface of ~15 μm, which is not an extrinsic defect, but seemingly a low strength matrix area. This could have been introduced to the material by poor CB networks as confirmed by TEM micrographs (Figure 3b). However, a rough fracture surface is visible indicating a slow crack growth throughout the crack propagation.

**Figure 8:**
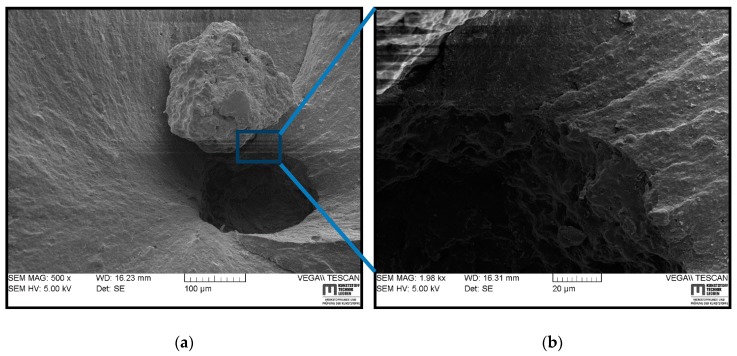
SEM images of a fracture surface of HNBR3 in different magnifications: (**a**) fracture initiation from a rigid body; (**b**) magnified view of fracture initiation and propagation.

SEM images of a fracture surface for HNBR3 are shown in Figure 8, where the crack initiated from a large cluster and as a result of the fracture (Figure 8a). The cluster was exfoliated from the surface creating a large pit on the surface and it could be a large CB agglomerate covered by the matrix or formed by other additives in the compound with a defect size ~150 μm. The Figure 8b magnifies the edge of the pit and the vicinity of the initiation point. Inside the pit, it seems that the matrix has failed around the large cluster, which was loosely bound. The vicinity of the initiation point was a rough and lamellar structure, which apparently indicates a stable crack growth. Therefore, it could be assumed that the gas is accumulated near the rigid inclusion during gas compression and upon decompression, the expansion of gas volume encounters the crack initiation and propagation through low-strength sites. The large inclusions behave as if they were smaller voids causing gas to accumulate, which helps a crack to grow and inflate under depressurization [34].

**Figure 9:**
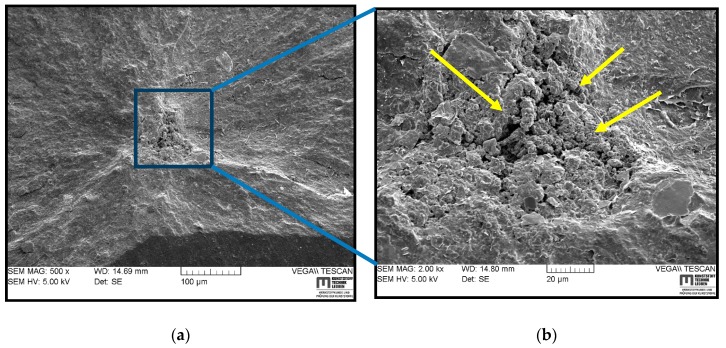
SEM images of a fracture surface starting from inherent voids of HNBR4 in different magnifications: (**a**) fracture initiation and propagation; (**b**) magnified view of fracture initiation and cracks into the material.

**Figure 10:**
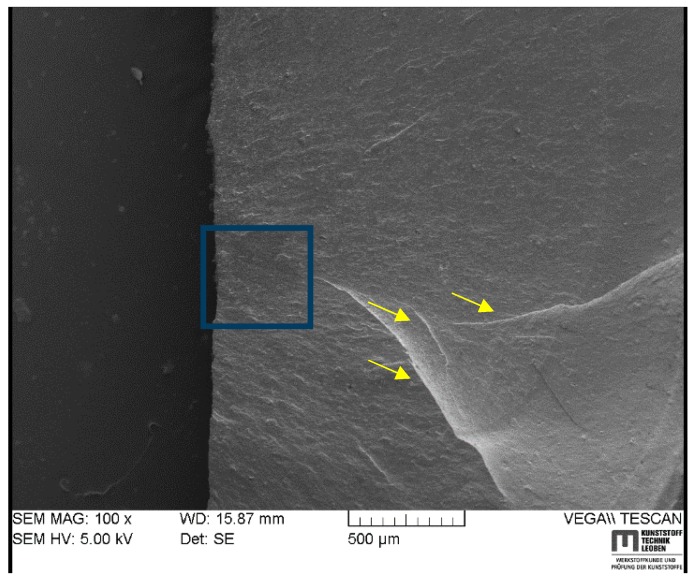
SEM image of a fracture surface starting from low-strength matrix site and a propagation in slow and subsequently fast stable crack growth for HNBR4.

Figure 9 and Figure 10 show two fracture surfaces of HNBR4 in different magnifications, which contain different crack initiation and propagation morphologies. Figure 9a displays an initiated crack inside the cylinder propagating to the outer surface, while Figure 9b magnifies the origin of the crack. As indicated by the arrows, the cracks were pronounced in the material in the fracture initiation region. This could be an indication that the fracture propagated from cavities, which were initiated as a result of inherent voids in the material. It can be assumed that the small voids in the material nucleated the gas during the pressurization, while during depressurization, they rupture, creating cavities in the material. These open cavities could act as stress raisers and offer an easy path to propagate flaws with cracks perpendicular to the cavities to a fatal fracture as many were observed in this material grade.

As shown in Figure 10, the initiation point (square highlighted) did not occur at a rigid inclusion or a cavity, but a flat surface, seemingly a low strength matrix surface. The crack propagation is different from other observed morphologies of the previous SEM images. As indicated in Figure 10a by arrows, there are hackle lines, which are formed by converging the small tearing lines to create a large crack. After the hackle lines, the crack propagation mode seemingly transitions from slow crack propagation to a stable fast crack. Therefore, a fast crack growth region is apparent with a rather flat, smooth surface compared with the slow crack propagation region. During the autoclave test, every HNBR4 specimen fell from the standing position and disappeared from the observation window due to this high-speed crack growth similar to a bursting behavior. The cracks were even visible to the naked eye on the outer surface of the tested cylindrical specimen.

The SEM analyses of the fracture surfaces of RGD tested samples revealed that the cracks initiated in the center region of the cylinder and propagated towards the circumference. The cracks were apparently initiated at the existing voids, hard body inclusions (particle agglomerate), or a low strength matrix site. Similar observations were reported in the literature for the RGD fracture initiation [3,6,7,12]. The small voids or hard inclusions are hard to avoid during processing [34], the low strength matrix sites are a result of lower filler distribution, uneven cross-link density or trapped rubber between large CB agglomerates which becomes ‘free’ once the aggregate structure breaks apart [7,12,24]. It was challenging to correlate the SEM fractography observations to the CB grade or size of the primary particles or agglomerates in different materials. However, HNBR1, HNBR2, and HNBR3 showed coarse surfaces, which contained small pits on the fracture surface, and it can be assumed to be traces of reinforcing agglomerates detached from the matrix. In HNBR4, a slow crack growth was transformed into rapid crack propagation to a catastrophic failure after the initial cracks converged into a critical size crack (hackle).

## 4. Summary and Conclusions

This work focused on the influence on rapid gas decompression failure of different grades of CB, which had different surface areas, filled in HNBR. Regarding the RGD test results, the trend of the volume increase of cylindrical specimens during the compression phase, which is a measurement of gas solubility into the material, evidently followed the component of CB with a lower surface area in the material. In general, filler loading reduces the elastically effective matrix component, increases mechanical properties of matrix, and decreases the gas sorption into elastomers. However, a poor filler–rubber interaction can trap the gas and increase sorption. In these highly filled conditions, the CB surface area has slightly influenced the solubility (assumed by compression phase volume increase) by reducing the effective matrix component and the increased mechanical properties. The tensile and DMA results proved the slight improvements in the mechanical properties based on higher CB surface area. However, HNBR4, which contained a greater amount of relatively higher surface area CB, revealed a rather different behavior compared with the other three grades. In addition, from the TEM micrographs, it can be assumed that HNBR4 contained bigger CB agglomerates and filler–filler networks. Therefore, a relatively higher stiffness at lower strain levels and softer material behavior at higher strain levels could be associated with the breakage of loosely bonded CB agglomerates and consequently, a rather free movement of molecules at higher strains. The volume increase in the decompression phase followed the same trend in compression phase in RGD tests, as a consequence of mechanical properties and the amount of saturated gas in the material, but NORSOK ranking showed a different rating. The initiated cracks can also possibly be responsible for some extent of lower volume increase during decompression phase. The TEM micrographs disclosed higher anisometric structures and better networks in samples with higher surface CB and relatively lower matrix components around them. HNBR2 showed the best RGD resistance next to HNBR3, where they retained lower CB structures as well as relatively greater matrix components around the CB structures. HNBR4 and HNBR1 disclosed relatively larger and higher number of cracks on the cross-sections after RGD. The greater filler structure discourages movement of rubber molecules around them from forming bound rubber and occluded rubber and it decreases effective matrix components. Therefore, HNBR1 lacks matrix material around the hard inclusions, which can elastically deform and assist the possible bubble nucleation and growth during the decompression phase. However, HNBR4, which contains a greater amount of CB agglomerates, eases crack growth after loosely bonded filler–filler bond rupture. Apparently, a minimum amount of matrix component around the CB grades is necessary to maintain the RGD resistance, along with other highly relevant parameters, as discussed above, for example, higher stiffness, tear, and tensile strength, lower gas solubility, etc. Therefore, in highly filled conditions, the size of the CB primary particles influences the properties in harsh environment utilization. The smaller CB particles tend to create denser filler networks and greater amount of agglomerates, which reduce the effective matrix component in the composite. Apparently, this is adversely effective in RGD resistance. SEM-based fractography did not influence the crack initiation based on different CB grades. However, it revealed that the cracks initiated from possible low strength matrix areas, hard inclusions or existing voids propagated to the outer surface.

## Figures and Tables

**Table 1:** The model hydrogenated nitrile butadiene rubber (HNBR) grades and their CB contents.

Grade	Content 1	Content 2
HNBR1	large size (10 phr)	middle size (75 phr)
HNBR2	large size (75 phr)	middle size (10 phr)
HNBR3	large size (40 phr)	middle size (45 phr)
HNBR4	large size (10 phr)	small size (75 phr)

**Table 2:** The seal-failure rating criteria according to NORSOK testing standard [30].

Description	Rating
No internal cracks, holes, or blisters of any size.	0
Less than 4 internal cracks, each shorter than 50% of the cross-section, with a total crack length less than the cross-section	1
Less than 6 internal cracks, each shorter than 50% of the cross-section, with a total crack length of fewer than 2.5 times the cross-section.	2
Less than 9 internal cracks of which max. 2 cracks can have a length between 50% and 80% of the cross-section.	3
More than 8 internal cracks, or one or more cracks longer than 80% of the cross-section.	4
Crack(s) going through the entire cross-section or complete separation of the seal into fragments.	5
